# Modeling Habitat Suitability for *Cerithidea rhizophorarum* and *Telescopium telescopium* in Indo‐West Pacific Mangroves

**DOI:** 10.1002/ece3.70384

**Published:** 2024-09-30

**Authors:** Hussaini O. Adamu, Rahimat O. Hussaini, Ebenezer O. Mohammed, Joshua I. Izegaegbe

**Affiliations:** ^1^ University of Bremen Bremen Germany; ^2^ City University of Applied Sciences (Hochschule Bremen) Bremen Germany; ^3^ University of Eastern Finland Kuopio Finland

**Keywords:** climate change, global species distribution, IWP, mangrove snails, MaxEnt modeling

## Abstract

Mangroves provide habitat for a diverse array of marine species, especially snails. We used a MaxEnt model to predict potential global suitable habitat for *Cerithidea rhizophorarum* and *Telescopium telescopium* in the family Potamididae. A total of 667 occurrence data were obtained from the Global Biodiversity Information Facility (GBIF) with the following sub‐data set contribution, “iNaturalist Research Grade Observations” (85%), “International Barcode of Life project (iBOL)” (7%), “FBIP: SeaKeys_SANBI: Marine images iSpot_2013” (1%), “A dataset of marine macroinvertebrate diversity from Mozambique and São Tomé and Príncipe” (1%), occurrence data of some marine invertebrates and freshwater crabs housed in the natural history collection at the National Museums of Kenya (1%), and Natural History Museum Rotterdam‐Specimens (1%). Our results showed that temperature with a contribution of above 80% in the present and future model is the most important driver of the distribution of mangrove snails. In the present and future models, the most potentially suitable habitats for *C*. *rhizophorarum* and *T*. *telescopium* were observed along coastal areas with a temperature between 20°C–21°C and 30°C, respectively. Our model predicts that by 2100, high‐suitability areas will shrink as a result of global warming. The vulnerability of mangrove snails under future climate conditions is evident in our results. Our findings contribute significant insights into the intricate relationship between mangrove habitats and mangrove snails, offering a valuable foundation for conservation initiatives aimed at safeguarding the biodiversity and ecological functions of these crucial coastal ecosystems in the face of changing global environmental conditions.

## Graphical abstract

Mangroves provide critical habitats for marine species, particularly snails. Using the MaxEnt model and 667 occurrence records from GBIF, we predicted suitable habitat for *Cerithidea rhizophorarum* and *Telescopium telescopium* in the Indo‐West Pacific. Temperature, contributing over 80%, was the key factor in both present and future models, with suitable habitats shrinking by 2100 due to global warming, highlighting the vulnerability of these species under future climate conditions.

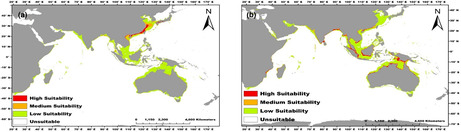

## 1 Introduction

Mangrove habitats are known to be associated with high biological productivity and rich biodiversity in tropical and subtropical regions (Macintosh, Ashton, and Havanon 2002; Kabir et al. 2014). Ecologically, mangroves serve as coastal protection from wind, currents, and waves and provide habitat, as well as feeding, nursery, and spawning grounds for various marine species (Vane et al. 2009; Getzner and Islam 2020).

Gastropods are one of the dominant invertebrate groups in the mangrove community and are known to play an important ecological role in the structure and function of mangrove ecosystems (Nagelkerken et al. 2008; Farahisah et al. 2023). Mangrove snail species such as *Cerithidea rhizophorarum* and *Telescopium telescopium* in the Potamididae family are some of the most abundant species found in the intertidal soft sediments of the Indo‐West Pacific (IWP) (Reid et al. 2008; Ota, Kawai, and Hashimoto 2013). Ecologically, *C*. *rhizophorarum* and *T*. *telescopium* feed on decomposing mangrove litter and other organisms that are attached to the roots and bark of mangroves contributing to the transport and degradation of organic matter in the intertidal ecosystem as well as serving as a source of food (protein) for local communities (Kabir et al. 2014; Ariyanto 2019; Adriman, Sumiarsih, and Andriani 2020; Farahisah et al. 2023). In addition, mangrove snails can be used to improve water quality. As biofilters, they can reduce levels of suspended matter and bacterial populations in wastewater from intensive shrimp farming effluents (Farahisah et al. 2023). Furthermore, the abundance and biodiversity of gastropods (mollusks) can serve as indicators of ecosystem health. Studies have shown that species from the genera *Cerithidea* and *Telescopium*, belonging to the Potamididae family, are effective bioindicators of ecological changes in mangroves and play a crucial role as decomposers in these ecosystems (Macintosh, Ashton, and Havanon 2002; Baderan et al. 2019).

The global oceans are changing due to climate change, impacting both biodiversity and habitats (Giangrande 2013; Hattab et al. 2014; Basher and Costello 2016). There are observable trends in how species respond to global warming, including a shift toward higher latitudes in oceans (Pinsky et al. 2013; Saeedi, Basher, and Costello 2017; Sharifian, Kamrani, and Saeedi 2021), increasing local extinction rates (Cheung et al. 2009), and alterations in local community compositions (Albouy et al. 2012). The use of species distribution modeling has provided valuable insights into predicting how various species will respond to climate change, both on land (Thuiller et al. 2008; Burrows et al. 2011) and in marine environments (Cheung et al. 2009; Albouy et al. 2013).

In recent years, one of the widely adopted techniques for modeling species distribution is the maximum entropy model—MaxEnt (Kramer‐Schadt et al. 2013; Weinert et al. 2016; Basher and Costello 2016; Rhoden, Peterman, and Taylor 2017; Drewnik, Węsławski, and Włodarska‐Kowalczuk 2017; Saeedi, Basher, and Costello 2017). This method relies on presence occurrence data and incorporates various predictor variables, including climatic and remotely sensed variables (Phillips and Dudík 2008; Sharifian, Kamrani, and Saeedi 2021). MaxEnt not only forecasts potential shifts in the future distribution range of species based on environmental and geographic data (Jetz, McPherson, and Guralnick 2012) but also assesses habitat suitability, potential future habitat loss, and potential invasion risks in response to climatic changes (Burrows et al. 2014). Predicted outcome models offer fundamental insights to improve conservation and species management initiatives (Saupe et al. 2014; Stuart‐Smith et al. 2015).

In this study, we utilized MaxEnt to model the present and future distributions of two ecologically important mangrove snails: *Cerithidea rhizophorarum* and *Telescopium telescopium* in the Indo‐West Pacific (IWP) for the first time. This involved incorporating depth, temperature, salinity, and current velocity as factors to predict potential shifts in the species distribution range in the future. Despite not being on the IUCN Red List, *C*. *rhizophorarum* is threatened with extinction in Japan, where habitat loss has made it vulnerable (Wada et al. 1996; Ota, Kawai, and Hashimoto 2013). Conversely, *T*. *telescopium* is considered a least‐concern species on the IUCN Red List and provides a food source in several Asian countries, thereby contributing to food security (Adriman, Sumiarsih, and Andriani 2020). Both species have significant ecological roles in the services they offer and the overall health of the ecosystem. Thus, it is important to understand the current and future species distribution and suitable habitats in the face of global changes.

## 2 Material and Methods

### 2.1 Species Distribution Data

The record of the global species distribution of mangrove snails belonging to the family Potamididae was obtained from the Global Biodiversity Information Facility (GBIF) with the following sub‐data set contribution, “iNaturalist Research Grade Observations” (85%; https://www.gbif.org/dataset/50c9509d‐22c7‐4a22‐a47d‐8c48425ef4a7), “International Barcode of Life project” (iBOL) (7%; https://www.gbif.org/dataset/040c5662‐da76‐4782‐a48e‐cdea1892d14c), “FBIP: SeaKeys_SANBI: Marine images iSpot_2013” (1%; https://www.gbif.org/fr/dataset/0b1150c9‐dfa8‐4904‐8bc5‐b79b4e1930e6), “A dataset of marine macroinvertebrate diversity from Mozambique and São Tomé and Príncipe” (1%; https://www.gbif.org/dataset/3e0e4ec9‐1905‐4cae‐9691‐c0fa79361ac3), occurrence data of some marine invertebrates and freshwater crabs housed in the natural history collection at the National Museums of Kenya (1%; https://www.gbif.org/dataset/6d2b478b‐5670‐4889‐a567‐8ae95f9b282e), and Natural History Museum Rotterdam Specimens (1%; https://www.gbif.org/dataset/a307e4d7‐1de2‐4adc‐95d5‐a0a8d5f57236).

Records lacking geographic coordinates, as well as those referring to land occurrences or fossil species, were excluded. Furthermore, the World Register of Marine Species (WoRMS) was used to verify the species names (David 2021; Maxim 2018), after which their synonyms and misspellings were corrected (Saeedi et al. 2019).

Our final dataset consists of 667 distribution records for *Cerithidea rhizophorarum* and *Telescopium telescopium* that belong to the genera (*Cerithidea* and *Telescopium*) with a record of ecological importance in the mangrove ecosystem and high sensitivity to environmental change (Wada et al. 1996; Macintosh, Ashton, and Havanon 2002; Ota, Kawai, and Hashimoto 2013; Baderan et al. 2019; Adriman, Sumiarsih, and Andriani 2020; Farahisah et al. 2023) (figure S1, table S1: https://doi.org/10.6084/m9.figshare.27021256).

### 2.2 Environmental Data

The environmental data used for our modeling were retrieved from Bio‐ORACLE (Tyberghein et al. 2012; Assis et al. 2017; Sharifian, Kamrani, and Saeedi 2021). These data were raster layers with a resolution of 5 arc‐min. We extracted the following pelagic layers, the average sea surface temperature (°C), salinity (PSS), and current velocity (m^−1^) for the present and future (2090–2100). The future environmental layer was extracted relative to 1986–2005 IPCC 2013 and RCP 8.5 as it is predicted that there will be a 4.8°C increase in global temperature in the future, and it will likely influence the latitudinal range of mangroves (IPCC 2014; Ward et al. 2016; Sharifian, Kamrani, and Saeedi 2021). Furthermore, we extracted data on depth (m), at 5‐arc‐min spatial resolution from the Global Marine Environment Datasets (GMED) (Basher, Bowden, and Costello 2018), with the assumption that depth (m) will remain constant until 2100 (Basher and Costello 2016; Saeedi, Basher, and Costello 2017; Sharifian, Kamrani, and Saeedi 2021). The relationship between environmental variables and the distribution of mangrove snails was evaluated with Pearson's correlation using the SDM tools box in ArcMap, removing all the variables with a correlation coefficient < 0.8 (Elith, Kearney, and Phillips 2010), and no relationship was observed between these variables (*p* > 0.05).

### 2.3 Modeling Approach

The modeling of the present and future distribution of mangrove snails was performed using the maximum entropy model (MaxEnt version 3.4.4) (Phillips, Anderson, and Schapire 2006). MaxEnt was configured using 10 cross‐validate replicate runs with 10,000 random background points. Thus, the average of the 10 predictions across all replicates was used for our analysis. In our models, we selected 75% of occurrence data for model training and 25% for model testing. The maximum iterations were set at 900 as suggested by (Dudík, Schapire, and Phillips 2006; Saeedi, Basher, and Costello 2017) and as applied by (Sharifian, Kamrani, and Saeedi 2021). The option “Remove duplicate records” in MaxEnt was used to avoid duplicate records within individual pixels of the background environment layers (Sharifian, Kamrani, and Saeedi 2021). The predicted areas with values below the minimum presence threshold (MPT) were considered environmentally unsuitable for the species.

### 2.4 Model Outputs and Interpretation

The model performance was evaluated using the area under the receiver operating curve (ROC) or AUC (Phillips, Dudík, and Schapire 2004). An AUC value of 0.5 indicates that the model did not perform better than random, whereas an AUC > 0.9 indicates perfect performance (Peterson et al. 2011). The percentage contribution of the variable and the permutation importance were used to investigate the relative relevance of the environmental variables. The relationship between environmental variables and the predicted probability of the presence of the two species was assessed using response curves. Based on the output of the logistic model of samples, the prospective species distribution was categorized into three groups using Natural Breaks (Jenks): high suitability (0.4–1), medium suitability (0.2–0.4), and low suitability (MPT—0.2) (Yang et al. 2013). This classification was used to interpret the suitability of the environment for mangrove snails.

The present and future habitat suitability, as well as the expected probability of species occurrence for mangrove snails, were mapped using ArcGIS 10.8.2 (ESRI 2018). The raster file containing the logistic output of MaxEnt for the probability of occurrence of each species was added to ArcGIS for both the present and future models. The probability values for presence in both the present and future models ranged from 0 to 1, where 0 represented no possibility of presence, intermediate values indicated a medium probability of presence, and 1 represented the highest probability of presence. In both the present and future models, the expected distribution of snails was mapped using only values close to 1, representing the highest probability of presence. To account for range changes between the present and future models, we used the merged SDMToolbox in ArcGIS to convert the present and future output raster files of *C*. *rhizophorarum* and *T*. *telescopium* to presence‐absence (1/0) format. We then subtracted the binary mapping of the future scenario from the present binary map.

## 3 Results

### 3.1 Model Performance

The models output performed well in predicting the distribution of *C*. *rhizophorarum* and *T*. *telescopium*. The AUC training values in the present and future model output for both species were < 0.98 (Table 1).

**TABLE 1:** Summary of MaxEnt outputs from present and future model outputs for *C*. *rhizophorarum* and *T*. *telescopium*.

Species	Training samples	Test samples	Iterations (present)	Training AUC ± SD (present)	Minimum presence threshold (present)	Iterations (future)	Training AUC ± SD (future)	Minimum presence threshold (future)
*Cerithidea rhizophorarum*	50.4	5.4	548	0.9941 ± 0.0031	0.0077	598	0.9939 ± 0.0031	0.0089
*Telescopium telescopium*	161.1	16.8	860	0.9868 ± 0.0038	0.007	844	0.9871 ± 0.0037	0.0037

### 3.2 Contribution of Environmental Variables

After checking for correlation between bioclimatic variables with SDM‐Toolbox, four bioclimatic variables, including temperature, depth, salinity, and current velocity, were used as the environmental variables for the prediction of *C*. *rhizophorarum* and *T*. *Telescopium*. The results revealed temperature, which contributed 70.1% and 60% for *C*. *rhizophorarum* and *T*. *Telescopium*, respectively, was the most important environmental variable driving the distribution of mangrove snails in both present and future conditions (figure S2; https://doi.org/10.6084/m9.figshare.27021256). This was followed by depth (present: 22.6%, future: 20.8%), salinity (present: 6.5%, future: 6.6%), and current velocity (present: 0.8%, future: 0%–9%) for *C*. *rhizophorarum*. For *T*. *Telescopium*, the various contributions include depth (present: 32.1%, future: 36.9%), salinity (present: 4.9%, future: 3.7%), and current velocity (present: 3.0%, future: 0.9%).

### 3.3 Mangrove Snails Response to Environmental Variables

The species response curve shows the relationship between environmental variables and the probability of species occurrence; they represent biological tolerance for species and habitat preference (Table 2).

**TABLE 2:** Response of *C*. *rhizophorarum* and *T*. *telescopium* to environmental variables.

Species	Present				Future			
	Temperature (°C)	Salinity (PSS)	Depth (m)	Current velocity (m^−1^)	Temperature (°C)	Salinity (PSS)	Depth (m)	Current velocity (m^−1^)
*C*. *rhizophorarum*	20–21	34	NVR	1.4–1.5	21	34	NVR	1.4–1.5
*T*. *telescopium*	30	29	19	0.8–1.0	30	28–29	19	0.8–10

*Note:* NR, No visible relationship.

### 3.4 Present Suitable Habitat

**FIGURE 1:**
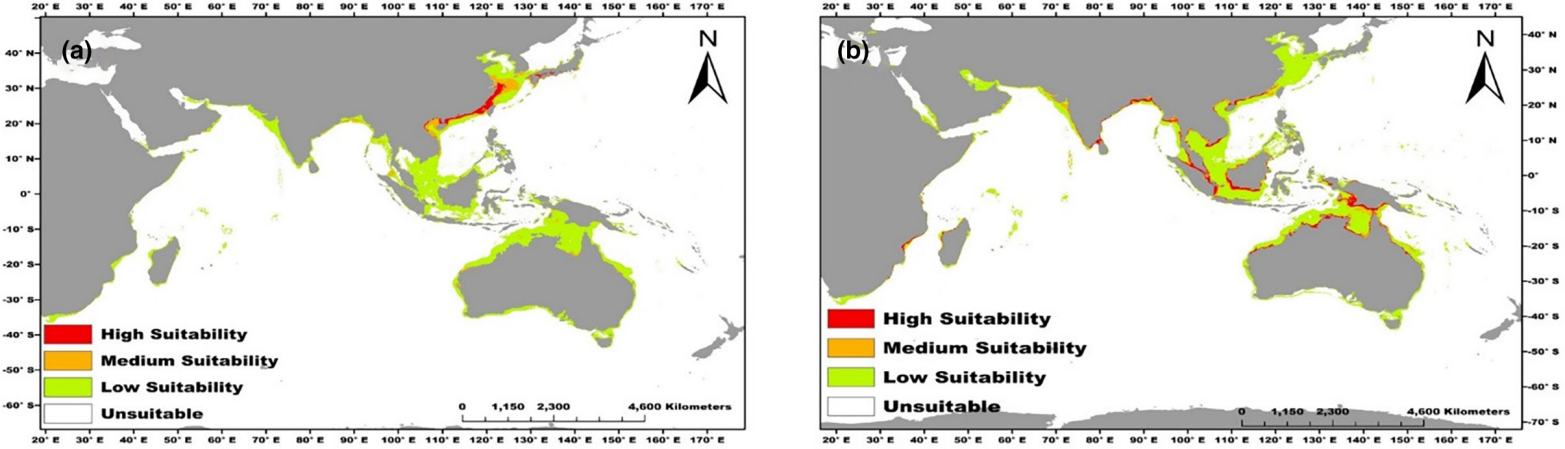
Predicted distribution points of *C*. *rhizophorarum* (a) and *T*. *telescopium* (b) under present environmental conditions.

We classified the species suitable habitat according to each species' MPT. The total suitable habitat with an area of 307.86 km^2^ for *C*. *rhizophorarum* was mainly located in Indonesia, Malaysia, Bangladesh, Madagascar, Thailand, Oman, Vietnam, China, Japan, and Australia (Figure 1a).

**FIGURE 2:**
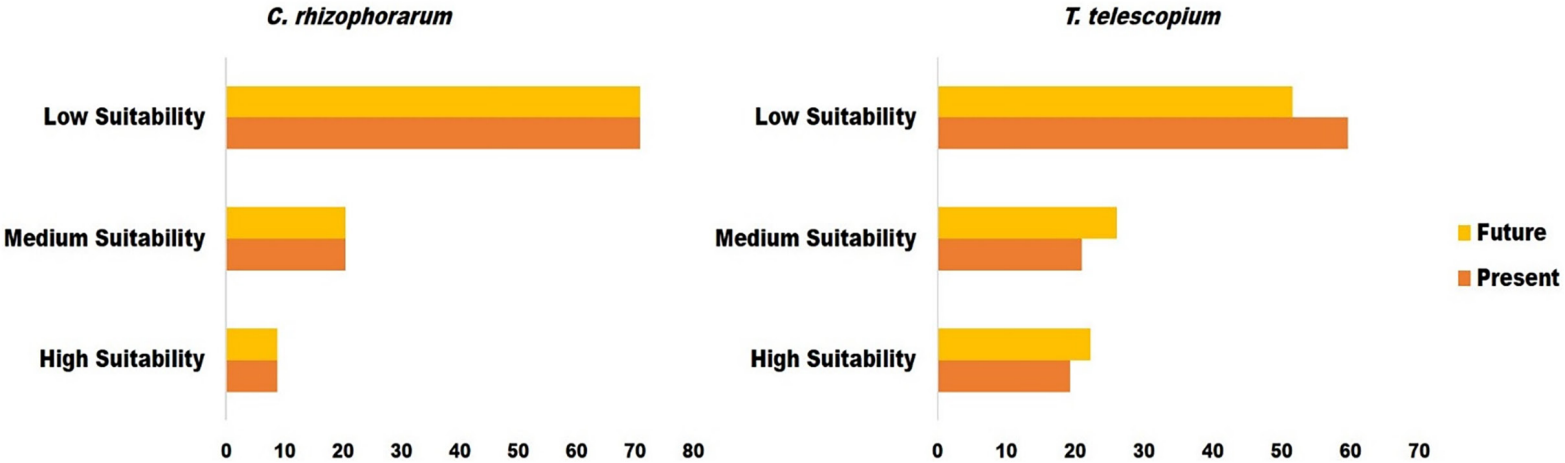
Variation in the area identified as suitable habitat in per cent for *C*. *rhizophorarum* and *T*. *telescopium* in the present and future models. High suitability, 0.4–1; medium suitability, 0.2–0.4; and low suitability, MPT—0.2.

Within the total suitable habitats, the high‐suitability habitat area for *C*. *rhizophorarum* was 26.75 km^2^ and accounted for 8.69% of the total suitable habitat. The highly suitable areas are China, India, Thailand, Japan, and Vietnam. The medium suitability habitat comprised 62.80 km^2^ and accounted for 20.40% of the total suitable habitat. The low suitability habitat comprised 218.31 km^2^ and accounted for 70.91% of the total suitable habitats area (Figure 2).

We obtained a total suitable habitat area of 405.02 km^2^ for *T*. *telescopium* occurring in coastal areas such as the Philippines, Oman, Papua New Guinea, South Africa, Mozambique, Madagascar, India, Bangladesh, Thailand, Indonesia, Australia, Vietnam, Japan, and Malaysia (Figure 1b).

The high‐suitability habitat area for *T*. *telescopium* was found to be 78.26 km^2^, which represents 19.32% of the overall suitable habitat. The countries with the highest suitability levels were Mozambique, Madagascar, India, Malaysia, Indonesia, Australia, Thailand, Vietnam, and China. The medium suitability habitat 85.12 km^2^ accounted for 21.02% of the total suitable area. The low suitability is made up of an area of 241.64 km^2^ and represents 59.66% of the total suitable area (Figure 1b and Figure 2).

### 3.5 Potential Suitable Habitats Under Future Climate

**FIGURE 3:**
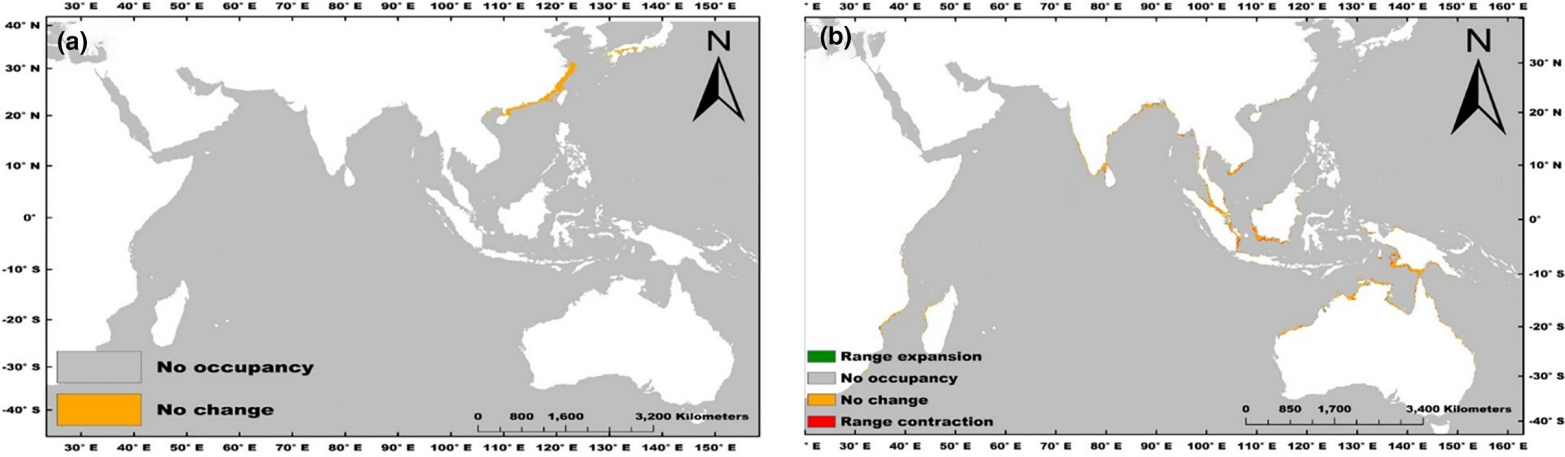
Changes in the suitable habitats of *C*. *rhizophorarum* (a) and *T*. *telescopium* (b) between future (year 2100; RCP 8.5) and the present climate conditions.

In the future (the year 2100), the total suitable habitat remains unchanged for *C*. *rhizophorarum* while for *T*. *telescopium* we recorded a range expansion and contraction of 23.44 and 64.29 km^2^, respectively (Figure 3).

**FIGURE 4:**
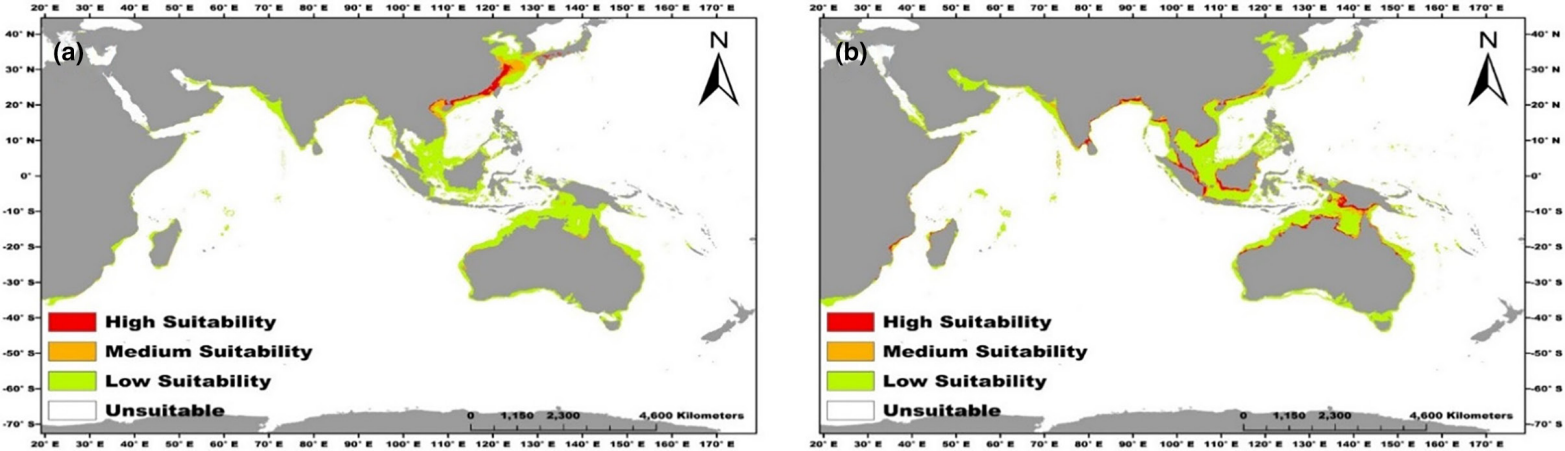
Predicted distribution points of *C*. *rhizophorarum* (a) and *T*. *telescopium* (b) under future climate; RCP 8.5 in the year 2100.

In the future, a high‐suitability habitat was observed for *C*. *rhizophorarum* in China, Vietnam, Japan, and India. (Figure 4a), while high‐suitability areas were recorded for *T*. *telescopium* in Australia, Malaysia, Papua New Guinea, Indonesia, Bangladesh, and India (Figure 4b). In the future model, the total area of suitable habitat (307.86 km^2^) as well as the classified suitability high, medium, and low suitability habitats for *C*. *rhizophorarum* is similar to the present model (Figure 2). The total suitable area recorded for *T*. *telescopium* was 325.79 km^2^, with a loss of about 79.23 km^2^ of the total suitable area in the future (Figure 2). In addition, the high‐suitability area accounted for 72.48 km^2^ (22.25%) of the total suitable area while the medium and low suitability accounted for 85.03 (26.10%) and 168.28 km^2^ (51.65%), respectively (Figure 2).

## 4 Discussion

### 4.1 Habitat Suitability of *C*. *rhizophorarum* and *T*. *telescopium* and Their Important Environmental Variables

The hypothesis that tropical ectotherms are at risk of physiological vulnerability to future environmental warming due to their present proximity to thermal limits has garnered substantial empirical backing (Tewksbury, Huey, and Deutsch 2008; Huey et al. 2009, 2012; Marshall et al. 2015). Furthermore, models calibrated with paleontological data predict elevated extinction risk in coastal tropical regions, particularly in response to human activity and climate change (Finnegan et al. 2015).

The findings of the present model predicated that mangrove snails thrive best in temperate and tropical coastal regions of North and South America, as well as the Indo‐West Pacific area. These locations are characterized by the highest reported density and diversity of mangrove forests and mangrove snails (Reid 1986, 2007; Snelgrove et al. 2016; Sharifian, Kamrani, and Saeedi 2021).

We found that the distribution of mangrove snails is primarily influenced by temperature, aligning with findings from previous studies. This is consistent with research on Antarctic benthic shrimps (Dambach et al. 2013; Basher and Costello 2016), Benthic Crustacea and Mollusca of Arctic fjord (Drewnik, Węsławski, and Włodarska‐Kowalczuk 2017), benthic marine invertebrates (Meißner et al. 2014), razor clams (Saeedi, Basher, and Costello 2017), endo helminth parasites (Kuhn, Cunze, and Kochmann 2016), mangrove snails (Farahisah et al. 2023), and future distribution of mangrove crabs in response to climate change (Sharifian, Kamrani, and Saeedi 2021).

Earlier research emphasized that temperature is a key factor influencing the distribution and diversity of marine species (Hattab et al. 2014; Meißner et al. 2014; Reiss et al. 2015). In this study, our results showed that temperature is responsible for the distribution of *C*. *rhizophorarum* and *T*. *telescopium*. This corresponds to the study of Hirata (1991), where temperature played a key role in the extended distribution range of *C*. *rhizophorarum*. In both present and future model outputs for *C*. *rhizophorarum* and *T*. *telescopium*, distribution was observed to be determined by temperature, while salinity, depth, and current velocity play no role in their distributions. Previous studies have reported that *T*. *telescopium* is an euryhaline species and can tolerate a wide range of salinity from 15 to 34 ppt, as well as desiccation for up to 6 months (Vermeij 1973; Alexander and Rae 1979; Ganguly et al. 2020).

### 4.2 Changes in the Distribution of *C*. *rhizophorarum* and *T*. *telescopium* in the Future

The models predicting the future distribution of mangrove snails indicate that temperature is a crucial factor in modeling the distributions of both species, aligning with findings from earlier studies (Dambach et al. 2013; Domisch et al. 2013; Basher and Costello 2016; Saeedi, Basher, and Costello 2017; Sharifian, Kamrani, and Saeedi 2021). The prediction of future suitable habitats indicated a higher percentage of low suitability habitats of about 43.62% and 55.27% for *C*. *rhizophorarum* and *T*. *telescopium*, respectively, compared to high suitability ones. This is likely due to the poleward shift in the mangrove range by at least 2° of latitude, resulting in a reduction in the availability of suitable ecosystems for mangrove snails (Record et al. 2013; Sharifian, Kamrani, and Saeedi 2021).

The mangrove snail occurrence data used for modeling were all in the Indo‐West Pacific realm, model output predicted a shifting distribution trend of *C*. *rhizophorarum* and *T*. *telescopium* from the Indo‐West Pacific realm toward its central areas and Atlantic East Pacific realms in the future, where Oman, East and South China, North and South America, and West Africa would be the most suitable habitat for mangrove snails in the future. Record et al. 2013, predicted a substantial concentration of mangrove species in some of these areas until the year 2080. The Indo‐West Pacific regions were predicted to be the most suitable habitats for mangrove snails in the future, a prediction consistent with other marine organisms, such as razor clams (Saeedi, Basher, and Costello 2017), mangrove crabs (Sharifian, Kamrani, and Saeedi 2021) and with the prediction for tropical mollusks (Saupe et al. 2014). Geographic barriers and regional topography have been identified as constraining factors in the future distribution of benthic marine organisms, compared to fish or plankton (Cosel 2002; Gallien et al. 2010; Stransky and Svavarsson 2010; Brix and Svavarsson 2010; Weinert et al. 2016). Although marine organisms exhibit rapid shifts in species distribution compared to their terrestrial counterparts, this is attributed to fewer dispersal barriers in marine environments and higher temperature adaptations of marine organisms as opposed to terrestrial species (Cheung et al. 2009; Burrows et al. 2011; Sunday, Bates, and Dulvy 2012).

Our findings suggest that temperature is the most effective predictor of the present and future distributions of mangrove snails (Hirata 1991; Meißner et al. 2014).

To show the global future distribution of mangrove snails due to climate change and to establish potentially suitable habitats, we modeled the global distribution of mangrove snails for the first time. Our results showed that the decrease in high‐suitability habitat due to global warming in the future confirms the role of temperature, which can be disturbing, especially for species with endemic and limited distribution ranges. Mangroves provide habitat for numerous ecologically and economically important organisms; given the above results, it is recommended that research be carried out on the remaining organisms that inhabit the mangrove ecosystem to determine how climate change will alter their distribution.

## Author Contributions

**Hussaini O. Adamu:** conceptualization (lead), data curation (lead), formal analysis (lead), methodology (equal), software (equal), validation (equal), writing – review and editing (equal). **Rahimat O. Hussaini:** conceptualization (supporting), formal analysis (supporting), visualization (equal), writing – original draft (equal). **Ebenezer O. Mohammed:** data curation (supporting), methodology (equal), writing – original draft (equal). **Joshua I. Izegaegbe:** data curation (supporting), methodology (supporting), software (supporting), writing – original draft (equal).

## Conflicts of Interest

The authors declare no conflicts of interest.

## Data Availability

The data have been deposited with Figshare: https://doi.org/10.6084/m9.figshare.27021256.
